# The Evolution of Tumor Microenvironment in Gliomas and Its Implication for Target Therapy

**DOI:** 10.7150/ijbs.83531

**Published:** 2023-08-21

**Authors:** Yang Hu, Zhixing Li, Yichi Zhang, Yuzheng Wu, Zihao Liu, Jianhao Zeng, Zhexue Hao, Jin Li, Jiaoyan Ren, Maojin Yao

**Affiliations:** 1 The First Affiliated Hospital of Guangzhou Medical University, Guangzhou Institute of Respiratory Disease & China State Key Laboratory of Respiratory Disease, Guangzhou, 510182, China.; 2 School of Food Sciences and Engineering, South China University of Technology, Guangzhou, 510641, China.; 3 Department of Microbiology, Immunology, and Cancer Biology, University of Virginia Health System, Charlottesville, VA 22908, USA.

**Keywords:** Glioma, Tumor microenvironment, Tumor evolution, Targeted therapy, Mouse model

## Abstract

Gliomas develop in unique and complicated environments that nourish tumor cells. The tumor microenvironment (TME) of gliomas comprises heterogeneous cells, including brain-resident cells, immune cells, and vascular cells. Reciprocal interactions among these cells are involved in the evolution of the TME. Moreover, the study of attractive therapeutic strategies that target the TME is transitioning from basic research to the clinic. Mouse models are indispensable tools for dissecting the processes and mechanisms leading to TME evolution. In this review, we overview the paradoxical roles of the TME, as well as the recent progress of mouse models in TME research. Finally, we summarize recent advances in TME-targeting therapeutic strategies.

## Introduction

Tumors are complex tissues that are composed of malignant cells and multiple distinct niche cells 1. In solid tumors, the tumor microenvironment (TME) comprises vasculature, extracellular matrix (ECM), non-malignant cells surrounding the tumor (including stromal cells, fibroblasts, and immune cells), and environmental molecules (such as growth factors, cytokines, chemokines, and exosomes). Bidirectional communication between tumor cells and their microenvironment has been revealed to be actively involved in tumor initiation, progression and even impede the efficacy of therapy. To better understand the evolution of the TME, the “seed and soil” theory was first proposed by Stephen Paget in 1863 to delineate the tropism of tumor metastases to specific organs 2. Since then, increasing evidence has confirmed the essential role of the TME in tumorigenesis 1. The TME provides a nourishing environment for every stage of cancer development, including primary tumor growth, metastatic dissemination and survival in the periphery, as well as secondary outgrowth 3.

For cancer treatments to be effective, the drugs must reach the tumor tissue at adequate concentrations. However, abnormal vasculature can limit the efficacy of drug delivery and treatment 4, and alteration of stromal cells may contribute to both inherent and acquired resistance 5. Hence, drugs targeting TME cells have been developed and clinically tested. Unlike tumor cells, stromal cells within the TME are genetically and/or epigenetically stable, and thus, targeting the TME is an even more attractive option with minimal risk of therapeutic resistance.

Brain tumors are one of the leading causes of cancer-associated mortality, with limited treatment options. According to the World Health Organization (WHO), primary brain tumors can be classified into more than 100 types based on phenotypic and genotypic parameters 6. Approximately 80.9% of primary malignant brain tumors are gliomas, of which glioblastoma multiforme (GBM) is the most aggressive type. Relative survival estimates for GBM are poor, with 5.6% of patients surviving five years post-diagnosis 7, and the median overall survival (OS) is only 14.6 months after receiving combination therapy of surgery, temozolomide chemotherapy and radiotherapy 8. Consequently, clinical treatment of brain tumors, especially GBM, remains a significant challenge.

The TME has critical effects on all stages of tumorigenesis, recurrence and therapeutic response. Tumor cells produce and secrete a variety of cytokines and growth factors, which promote the infiltration of non-neoplastic cells and alter their physiology toward pro-tumorigenic features. Compared with the TME in other cancers, the brain TME is distinctive because it contains several unique tissue-resident cell types, including microglia, astrocytes and neurons. In addition, the blood-brain barrier (BBB) or blood-brain tumor barrier (BBTB), formed by endothelial cells, pericytes and astrocytic endfeet, shields the brain from immune cell entry and/or attack 9. The majority of non-neoplastic cells within gliomas are tumor-associated myeloid cells (TAMs), accounting for up to 30% of the tumor mass, and can originate from both brain-resident microglia and bone marrow-derived macrophages (BMDMs) 10. In addition, astrocytes 11 and neurons 12 are both brain-specific cell types that are tightly involved in the initiation and progression of gliomas. Due to its crucial role in gliomas, the TME may be a therapeutic target to improve outcomes for patients. Most importantly, tumor cells co-evolve with TME cells from tumor initiation to progression along with the increased complexity. In this review, we will discuss the roles of these components and the potential reciprocal interactions involved in TME evolution.

## Tumor initiation and TME development

In the last century, it has been well recognized that cumulative somatic mutations drive tumorigenesis. Fearon and Vogelstein 13 first hypothesized a genetic model for colorectal tumorigenesis in which mutations occur, accumulate, and contribute to the transformation of normal epithelial cells to malignant cells. Hanahan and Weinberg 1, 14 further conceived that eight essential alterations in cell physiology serve as hallmarks of cancer. However, ostensible niche cells recruited by cancer cells contribute to the acquisition of these hallmarks and exert influences on cancer development by creating the TME 1.

Tumor initiation might require a distinct microenvironment since mutant cells can constantly shape the surrounding niche. Cancers are not just clones of mutant and malignant cells but complex abnormal organs, to which multiple normal cell types are recruited by malignant cells under changes that promote tumor development. Bidirectional communication between cancer cells and their surrounding environment creates and ignites the evolution of the TME. The steps of tumor formation involve the co-evolution of neoplastic cells and components of the surrounding stroma. However, the mechanisms underlying the infiltration of tumor cells into the TME and the interaction of TME components with tumor cells remain unclear. The following sections aim to elucidate the cellular and molecular mechanisms in gliomas.

## Cellular components in glioma

### Tumor-associated myeloid cells (microglia/macrophages)

**Figure 1:**
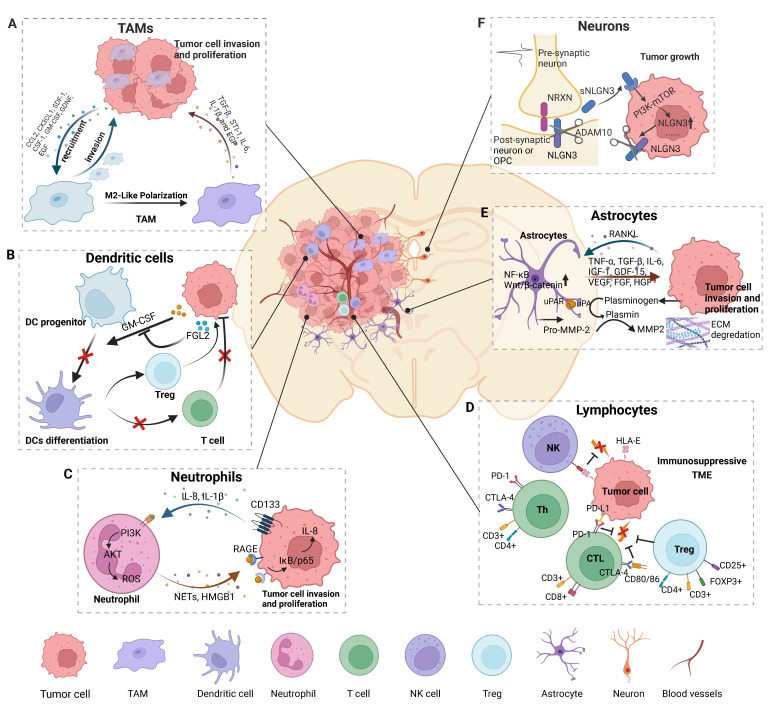
** Cell components and their significant roles in the glioma microenvironment.** The brain TME comprises cells of the immune system, including various specialized organ-resident cell types. Tumor formation involves the co-evolution of neoplastic cells and surrounding non-transformed cells. The TME is both a cause and consequence of tumorigenesis. A) Tumor cells release factors to recruit TAMs into the microenvironment and polarize TAMs to an M2-like phenotype, and TAMs in turn promote glioma progress. B) Glioma secretes factors to inhibit DC differentiation and subsequently causes a lack of CD8+ T cell priming and activation, and an increase in the number of Tregs results in the development of the TME of glioma. C) NETs mediate the crosstalk between glioma cells and neutrophils by regulating the HMGB1/RAGE/IL-8 axis. D) The cytotoxic capacity of NK cells is reduced due to the suppression of HLA-G from GBM cells. CD8+ and CD4+ T cells highly express genes involved in cell exhaustion, creating an immunosuppressive microenvironment that favors tumor growth, which is regulated by regulator T cells. E) Tumor cells release cytokines to activate astrocytes and TAAs, which in turn promote glioma growth and invasion by releasing pro-tumor factors and increasing ECM degradation. F) Neuronal activity-dependent secretion of the synaptic NLGN3, cleaved by ADAM10 sheddase, promotes tumor cell proliferation by activating the PI3K-mTOR pathway. *Figures were created with biorender.com.*

The predominant immune cellular components in gliomas are TAMs (Figure 1A), which account for up to 30%-50% of the tumor mass 15. TAMs can be subdivided ontogenetically into at least two major populations: (1) tissue-resident microglia (TRMs) of yolk sac origin and (2) BMDMs. As tissue-resident macrophages, microglia act as the first and main form of active immune surveillance in the central nervous system (CNS). Once inflammation is triggered, microglia can rapidly migrate toward the brain lesion, where they phagocytose and remove debris and release cytotoxic molecules such as proinflammatory cytokines, reactive oxygen intermediates and proteinases. However, this antitumor effect can be attenuated when microglia are reprogrammed in the setting of glioma. In the presence of microglia, the migration and invasiveness of glioma cells was increased *in vitro* 16. TAMs depletion by infusion of ganciclovir in CD11b-HSVTK mice largely attenuated glioma expansion 17. Several factors, including STI1, EGF, CSF-1, CCL2, IL-6, TGF-β and TGF-β2, are released from TAMs and can promote glioma proliferation and/or migration 10. Some flow cytometry experiments have demonstrated that the majority of TAMs in gliomas are derived from monocyte-derived macrophages but not resident microglia 18. Another study using irradiation chimeras revealed that intrinsic microglia are the main source of brain tumors 19. Elucidation of the cellular origin of TAMs could provide new therapeutic strategies aiming to inhibit the recruitment of monocytes from blood. Moreover, redirecting their phagocytosis function to sustain their “defender” antitumor activity was proposed as a new strategy to target TAMs 20. Of note, Rao et al. showed that microglia and macrophages rather than CD8^+^ T cells mediated the therapeutic effect of PD-1 blockade 21. Understanding the precise nature of TAMs is critical for not only improving the understanding of the role of TAMs in cancer biology but also identifying ways to better exploit macrophage/microglia-based therapy in the clinic.

### Dendritic cells

Dendritic cells (DCs) are professional antigen-presenting cells and play a critical role in both the innate and adaptive immune responses (Figure 1B). DCs can be divided into two distinct subsets: myeloid DCs (mDCs) and plasmacytoid DCs (pDCs). pDCs promote glioma progression in a murine model, while the depletion of pDCs increases the survival of mice bearing glioma by decreasing the number and suppressive function of regulatory T cells (Tregs) 22. Furthermore, TGFβ and IL-10 secreted by glioma cells can disrupt the normal function of DCs. In glioblastoma, FGL2, secreted from tumor cells, blocks GM-CSF-induced CD103^+^ DCs development by suppressing NF-κB, STAT1/5, and p38 activation. Subsequently, CD8^+^ T cells are not primed or activated, which results in GBM progression 23.

### Neutrophils

Neutrophils account for 50% to 70% of circulating leukocytes and are involved in almost all processes in tumor development (Figure 1C). Human glioma tissues have a high amount of neutrophil infiltration, and neutrophils are an important prognostic factor in glioma 24. Wang et al. found that glioma patients with higher levels of neutrophils or neutrophil to lymphocyte ratios were more likely to have poor prognosis 25. Neutrophil extracellular traps (NETs), produced by tumor-infiltrating neutrophils (TINs), are an oncogenic marker of high-grade gliomas (HGGs) and promote tumor cell proliferation and invasion 26. Furthermore, neutrophil count is a prognostic indicator for patients with IDH wild-type GBM treated with temozolomide 25. Increased neutrophil infiltration into tumors during anti-VEGF therapy promotes glioma progression and treatment resistance by increasing glioblastoma-initiating cell proliferation and migration, which is mediated by S100A4 27. Therefore, agents targeting neutrophils and S100A4 combined with standard antiangiogenetic therapy may inhibit glioma progression and diminish antiangiogenic therapy resistance. A growing number of studies have focused on the mechanism of neutrophil recruitment. Glioma cells ectopically expressing CD133 increase neutrophil recruitment via the IL-1 signaling pathway both *in vitro* and *in vivo*, indicating that CD133-positive tumor-initiating cells might generate a unique TME through co-evolution with neutrophils 28. IL8 is also an effective chemoattractant that recruits neutrophils at the tumor site and facilitates the proliferation of glioma cells 26. TINs have been found to mediated NETs formation and HMGB1 production via the PI3K/AKT/ROS axis. HMGB1, an important component of NETs, binds to RAGE and stimulates the NF-κB signaling pathway in tumor cells, thus promoting IL8 secretion and glioma progression 26. Neutrophils have an inherent ability to traverse the BBB/BBTB and penetrate the glioma site. Surgical tumor removal leads to the release of inflammatory factors, such as IL-8, which activate neutrophils and cause them to migrate to the glioma. Therefore, neutrophil-mediated drug delivery systems are an ideal treatment approach due to their enhanced therapeutic specificity and efficacy in glioma. Xue et al. reported a novel preclinical antitumor therapy strategy to eliminate residual glioma cells by delivering drugs into tumor lesions using a system mediated by neutrophils and paclitaxel-loaded liposomes 29.

### Lymphocytes

Lymphocytes primarily comprise CD4^+^ and CD8^+^ T cells, a small proportion of B cells and natural killer (NK) cells (Figure 1D). CD8^+^ T cells are considered critical for tumor clearance, and increased tumor-infiltrating CD3^+^ and CD8^+^ T cell numbers are associated with prolonged patient survival in glioblastoma 30. During tumor evolution, brain cancer cells develop strategies to escape T-cell antitumor responses. Glioma cells inhibit immune activation by secreting TGF-β as well as IL-10 and downregulating MHC class II expression on monocytes 31. PD-L1 is one of the most prominent inhibitory regulators of lymphocytes. Higher expression of PD-L1 predicts a poor prognosis in glioma patients 32. Glioma cells can upregulate PD-L1 expression in circulating monocytes and TAMs through autocrine/paracrine IL-10 signaling 33. CTLA-4 is another immune checkpoint protein that negatively regulates T-cell activation. Higher CTLA-4 expression is detected in high-grade glioma samples and is caused by a lack of CD80/86 co-stimulatory molecule expression 34. Chongsathidkiet et al. found that the number of circulating T cells in patients with glioma was less than 1/3 of that of normal people, and the T cells that were lacking from circulation were found sequestered in bone marrow. After hindering S1P1 internalization, T-cell sequestration is restored 35. This may partly explain why most gliomas are “cold tumors”, with less T cell infiltration in and around the tumor lesion. CD4^+^CD25^+^FoxP3^+^ Tregs are a pro-tumorigenic subpopulation because of their highly immunosuppressive effects in many cancer types (Figure 1D). Factors such as proliferating and dying tumor cell self-antigens can recruit Tregs around tumor lesions. Of note, tumor cell- and/or DC-derived TGF-β can greatly induce the enrichment of Tregs. Soluble factors, such as CCL22 or CCL2, secreted by glioblastoma cells attract Tregs to the local tumor environment 36, 37. The accumulation of Tregs is an important immune escape mechanism for gliomas. Heimberger AB et al. found that different amounts of Tregs do not seem to be related to patient outcome 38. However, another study showed that increased Treg frequencies were associated with early tumor recurrence and reduced survival rates in glioblastoma patients 39. Treg activity can be modulated to combat immunosuppression and enhance the anti-tumor immune response in these patients. Systemic anti-CD25 administration hindered the suppressive function of CD25^+^ Tregs, while it greatly enhanced the CD8+ cytotoxic T-cell response 40.

NK cells are another kind of cytotoxic lymphocyte that can identify and kill tumor cells and infiltrate different gliomas (Figure 1D). Research has shown that NK cells can recognize and eradicate stem cell-like human glioblastoma cells that express low amounts of HLA-I molecules and express several ligands recognized by activating NK receptors 41. Although NK cells have potent cytotoxic effects on medulloblastoma cells through the NKG2D/MICA-ULBP-2 interaction, high expression of HLA-I can protect medulloblastoma cells from the cytotoxicity of NK cells 42. Most studies to date have focused on the interaction of NKG2D with its ligand HLA-E and methods for restoring the NK cell-mediated anti-tumor immune responses.

### Vasculature

#### The BBB and angiogenesis

The blood vessels are mainly lined with tightly packed endothelial cells surrounded by pericytes. In the brain, these cells together with astrocytes form the BBB. The BBB functions as a selective physical barrier between the brain and the periphery that distinguishes the brain from other organs 43. As it restricts the transportation of large, hydrophilic molecules, the BBB protects the brain from toxins, pathogens and inflammatory cells. This characteristic also hinders the delivery of therapeutic agents into the brain, which is the factor limiting clinical use. However, the integrity of the BBB is often disrupted during tumor progression, resulting in intravascular leakiness. Recent research has shown that invasive glioma cells can displace astrocytic endfeet from endothelial or vascular smooth muscle cells, causing local damage to the BBB 44. However, not all brain tumor types or subtypes show the same degree of BBB impairment. For example, compared with SHH-medulloblastoma, WNT-medulloblastoma has a more permeable vasculature which enables the accumulation of chemotherapeutic drugs within the tumor lesion 45.

The tumor-associated vasculature within the brain has abnormal endothelial walls, pericyte coverage and basement membranes 46. The leakiness of tumor vessels causes an abnormal blood flow, increased interstitial fluid pressure and edema, which block effective delivery of therapeutic agents within tumors. Vascular endothelial growth factor (VEGF) is highly expressed in gliomas and promotes vascular permeability, extracellular matrix degeneration and vascular endothelial cell migration, proliferation, and angiogenesis. VEGF expression can be upregulated via hypoxia-dependent mechanisms and hypoxia-independent mechanisms such as PI3K-Akt, MAPK and Raf signaling. High VEGF leads to immature, dysfunctional vessels and an impaired BBB. Targeting blood vessel-related factors, particularly targeting VEGF and VEGFR, has been an attractive strategy in gliomas.

#### Lymphatic vessels

In the past, it was commonly believed that the lymphatic drainage system was notably absent from the brain. However, recent research has indicated that the meninges have a lymphatic vascular network, that is, the meningeal lymphatic system. Meningeal lymphatic vessels (MLVs) are connected to the deep cervical lymph nodes (CLNs) and efficiently drain fluid, soluble molecules and immune cells from the cerebrospinal fluid into CLNs 47, 48. Recently, study showed that the presence and distribution of new lymphatic vessels in glioma samples via immunohistochemical staining for the marker molecules CD31, Prox1, and LYVE-1 49. Intracranial tumors in mice induce extensive remodeling of dorsal MLVs 50. Disruption of dorsal MLVs blocks DC trafficking from brain tumors to deep CLNs and reduces the efficacy of combined anti-PD-1/CTLA-4 checkpoint therapy 50. Furthermore, VEGF-C, an important lymphangiogenic factor, may potentiate checkpoint therapy. Mice bearing tumors overexpressing VEGF-C respond better to anti-PD-1/CTLA-4 combination therapy, and these effects are abolished by either dorsal MLV ablation or CCL21/CCR7 blockade 50. Hence, dorsal MLVs might be a potential target in glioma immunotherapy.

### Astrocytes

Astrocytes account for approximately half of all brain cells and play an important role in the CNS (Figure 1E). These cells are components of the BBB and are responsible for mediating ionic and transmitter homeostasis, modulating synaptic activity and plasticity, and responding to CNS damage 11, 51. Immunohistochemical studies of human glioma biopsies have revealed that reactive astrocytes are located around gliomas 52. These reactive astrocytes may interact with glioma cells and contribute to tumor growth and aggression (Figure 1). Tumoral RANKL activates astrocytes via the NF-κB signaling pathway, leading to an increase in tumor-associated astrocytes (TAAs). These TAAs in turn secrete various factors, such as TGF-β, which promote glioma cell invasion 53. In the same way, glioma cells significantly activate astrocytes by upregulating Wnt/β-catenin signaling, and then astrocytes increase the degradation of ECM to promote tumor invasiveness. 54. TAAs can secrete TNF-α, TGF-β, IL-6, IGF-1, GDF-15, VEGF, FGF, HGF, and EGF to enhance the proliferation of GBM cells 11, 51. Moreover, TAAs might also participate in the adaptation of GBM cells to a hypoxic microenvironment through CCL20/CCR6 signaling, thus promoting angiogenesis and tumor cell invasion 11. In addition, astrocytes have been shown to be involved in resistance to radiotherapy or chemotherapy 55. Multiple factors, such as tenascin-C, IL-10, IFN-γ, IL-6, STAT-3, GDF-15 and PD-L1, secreted by TAAs protect GBM cells against anticancer immune reactions 51. Overall, modulating underlying TAA-associated signaling might be a promising approach for GBM treatment.

### Neurons

Neurons, a brain-specific cell type, are a crucial component of the brain TME and may contribute to the initiation and progression of tumors (Figure 1F). Active neurons exert a mitogenic effect on normal neural precursor and oligodendrocyte precursor cells (OPCs), the putative cells of origin of HGG 56-58. Optogenetic control of cortical neuronal activity promotes HGG cell proliferation and growth via the secretion of the activity-regulated mitogen synaptic protein neuroligin-3 (NLGN3) in a patient-derived pediatric HGG orthotopic xenograft model 59. Soluble NLGN3, released from post-synaptic neurons, promotes feedforward increases in the expression of NLGN3 through activation of the PI3K-mTOR pathway in glioma cells 59. Further research has demonstrated that the ADAM10 sheddase cleaves NLGN3 from both neurons and OPCs 60. Administration of an ADAM10 inhibitor robustly blocks HGG xenograft growth by preventing the release of NLGN3 into the TME in mice 60. Synapses exist between neurons, normal OPCs and other subpopulations in gliomas that closely resemble OPCs 61, 62. Monje et al. further found that structural synapses formed between glioma cells and neurons in the HGG microenvironment 63. Neuronal activity evoked glioma excitatory postsynaptic currents mediated by AMPAR and non-synaptic prolonged glioma potassium currents amplified by gap junction-coupled, and then induced glioma cell membrane depolarization 63. Glioma membrane depolarization induced by optogenetics promoted glioma xenograft cell proliferation, and pharmacological or genetic blockade of electrochemical signaling inhibited glioma xenograft growth 63. These findings illustrate the unexplored potential of targeting neural circuits for therapy and may provide promising new approaches for the development of therapeutics for the treatment of this devastating group of neural cancers.

### Cancer stem cell

Many researchers have identified and isolated glioma stem cells (GSCs) in GBM. GSCs promote tumor angiogenesis through elevated expression of VEGF 64. These GSCs are associated with vascular niches and interact with endothelial cells to regulate their self-renewal and tumorigenicity 65. Recently, Shideng Bao et al. showed that GSCs transdifferentiate into pericytes to support vessel function and tumor growth in xenograft models 66. GSCs are recruited to endothelial cells by the SDF-1/CXCR4 axis and generate vascular pericytes by TGFβ signaling 66. Selective elimination of GSC-derived pericytes (G-pericytes) by HsvTK-induced ganciclovir toxicity disrupts tumor vascular structure and function, and inhibits GBM growth 66. Moreover, targeting G-pericytes disrupts the blood-tumor barrier (BTB) and increases vascular permeability by impairing BTB tight junctions, which enhances drug delivery to improve GBM chemotherapy efficacy 67. BMX is an essential factor for maintaining G-pericytes. Inhibiting BMX with ibrutinib disrupts G-pericytes and the BTB to increase vascular permeability, effectively improves GBM treatment efficacy and extends the survival of tumor-bearing mice 67.

## Functional importance of TME cell types for glioma progression

**Figure 2:**
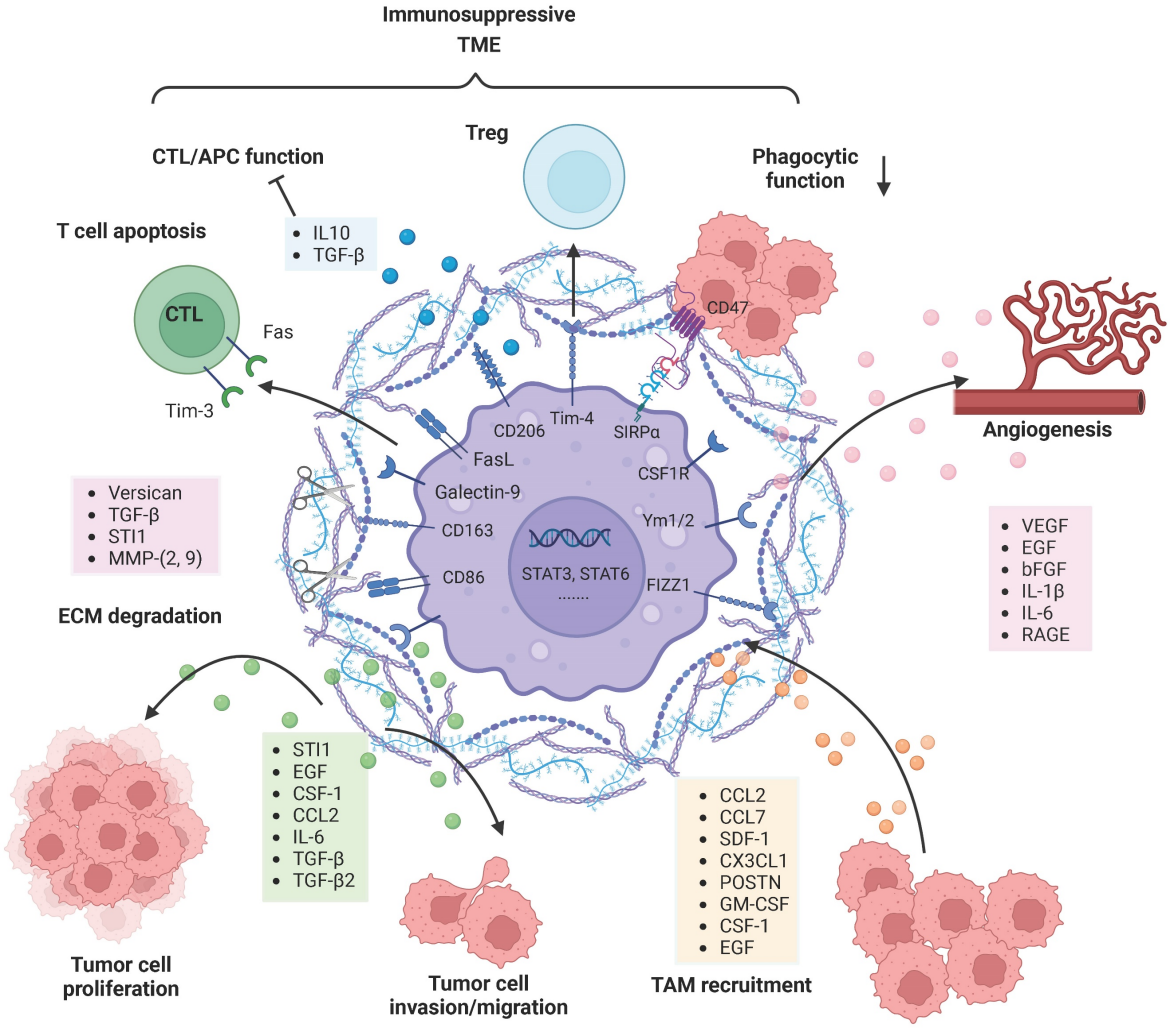
**TAMs reciprocally interact and co-evolve with tumor cells.** Tumor cell-derived factors recruit and polarize TAMs into an anti-inflammatory and pro-tumorigenic M2-like phenotype, and TAMs substantially promote tumor growth, tumor cell migration, and invasion in diverse manners. TAM-derived factors can facilitate the destruction of the ECM and glioma vascularization. Moreover, M2-polarized TAMs contribute to an immunosuppressive tumor microenvironment by secreting soluble factors and via direct cell-cell interactions. *Figures were created with biorender.com.*

Many studies have revealed that TAMs are crucial for glioma progression (Figure 2). Chemoattractants such as MCP-1 (CCL2), MCP-3 (CCL7), SDF-1 (CXCL12), CX3CL1, POSTN, GM-CSF, M-CSF (CSF-1) and EGF produced by glioma cells actively recruit TAMs and thus promote tumor growth 68. CCL2 is one of the most important chemoattractants and was originally isolated from a human glioblastoma cell line. Elevated expression of CCL2 contributes to the accumulation of TAMs and promotes tumor aggressiveness in human glioma tissue 69. However, a recent study showed that CCL7 has a stronger correlation than CCL-2 with TAM infiltration in human gliomas 70. In a murine astrocytoma model, the secretion of SDF-1 by glioma cells specifically promotes tropism toward hypoxic tumor areas 71. CSF-1 is a chemokine that mobilizes monocytes toward sites of inflammation. The production of CSF-1 by glioblastoma cells attracts microglia and transforms them into a pro-tumorigenic phenotype, and microglia in turn stimulate glioblastoma cell invasion via EGFR activation 72. In summary, these chemoattractants secreted by glioma cells have potential as future therapeutic targets.

Once TAMs are recruited to the glioma milieu, they undergo significant *in situ* reprogramming in the glioma microenvironment, and unique gene expression patterns are acquired. For instance, genes encoding cell cycle-related proteins, Th17 immune response-related proteins, complement-related factors, ECM components, proteases, lipid metabolism mediators and clotting factors were upregulated in both BMDMs and microglia. Re-educated TAMs promote glioma cell invasion and tumor growth in diverse manners (Figure 2). TGF-β is mainly derived from TAMs and can actively facilitate glioma cell invasion. Induction of MMP-2 and MMP-9 expression by TGF-β promotes the invasive properties of glioma cells, possibly via degradation of the ECM 73. STI1 synthesized and secreted by microglial cells has been shown to promote tumor cell proliferation and migration through MMP-9 74. Versican is a modular proteoglycan released from glioma cells and is involved in the control of cellular growth and differentiation 75. TLR2 signaling in TAMs is substantially activated by glioma-derived versican, which leads to increased microglial MT1-MMP expression 76. Upregulated MT1-MMP expression triggers MMP protein release and enables the degradation of the ECM to promote glioma invasion.

Other studies have also demonstrated that TAM-derived factors such as VEGF, EGF, bFGF, IL-1β and IL-6 are able to facilitate glioma vascularization (Figure 2). Notably, the interaction between RAGE and its ligands is crucial during angiogenesis. Chen et al. revealed that RAGE signaling in TAMs drives angiogenesis and that RAGE ablation results in decreased expression of IL6 and VEGF and abrogates tumor angiogenesis 77. Furthermore, selective depletion of resident microglia induces similar effects, suggesting that resident microglia rather than peripheral macrophages are responsible for the pro-angiogenic effects 78. Overall, many TAM-derived substances are able to support and facilitate glioma maintenance.

In addition, TAMs contribute to an immunosuppressive TME through a variety of mechanisms (Figure 2). Although educated TAMs have high levels of antigen presentation-related gene expression, they do not exert antitumor effects by activating effector T cells. SIRPα is expressed on TAMs and activated by CD47, an anti-phagocytic “don't eat me” surface protein expressed on cancer cells, particularly cancer stem cells. The phagocytic function of TRM-derived TAMs is attenuated due to increased SIRPα-CD47 signaling 79. The immune checkpoint receptor PD-1 and its ligand PD-L1 play an important role in inhibiting the immune response. TAMs are the predominant source of PD-L1, and they greatly impair anti-tumor immunity. In addition, Rao Ganesh et al. revealed that the anti-PD-1 therapy may be mediated by cells of the innate immune system, especially TAMs, rather than CD8+ T cells 21. Another relevant immune checkpoint receptor is T-cell immunoglobulin and mucin-domain-containing molecule 3 (Tim-3), which is expressed on T cells, NK cells and DCs, and its binding to Galectin-9 results in the apoptosis of these cells. Feng Yuan et al. have found a strong correlation between Galectin-9 expression and M2 TAMs levels, suggesting the role of TAMs in regulating Tim-3 signaling 80. FasL is expressed on almost all TAMs and can induce activated T-cell apoptosis by binding to the Fas receptor 81. TAMs as well as DCs are able to increase STAT3 activity and thus stimulate the secretion of IL-10, TGFβ and other anti-inflammatory molecules 80. Meanwhile, the production of IFN-γ and IL-2 is significantly decreased, leading to effector T-cell unresponsiveness 82. Tregs are able to inhibit the actions of effector lymphocytes. Friederike et al. revealed that MHC class II(+)CD40(dim)CD86(dim)IL-10(+) microglia are potent inducers of Foxp3^+^ Tregs in a murine model 83. In addition, TIM4 is a newly described molecule expressed on TAMs. TIM4-expressing macrophages phagocytose apoptotic T cells in glioma and gain immune tolerogenic properties, further facilitating the development of Tregs 84. Further research is required to uncover the complex multidirectional interactions in the glioma microenvironment.

Recognition of these concepts will benefit our understanding of cancer biology and lead to the development of alternative strategies to treat human cancer.

## Glioma mouse models

Mouse models are the indispensable tools for understanding the composition of TME and the influence on disease, and developing effective therapies targeted to TME. Most common gliomas experimental models can be divided into xenograft transplantation models and genetically engineered models. Next, we discuss existing types of gliomas models, as well as the advantages and drawbacks of these models, to aid the choice of an appropriate animal model for specific experimental purposes.

### Transplantation models

The existing *in vivo* models include models generated from transplantation of established glioma cell lines or cells isolated from patient samples, representing the simplest approach to generating tumors and the least technically challenging. There are two types of transplantation models: allograft and xenograft models. Tumor growth and drug efficacy can be measured in models in which tumor cells are implanted subcutaneously. However, these models lack the brain TME. Consequently, orthotopic implantation is a superior approach for glioma research. Xenograft models are generated via intracranial injection of human glioma cells (e.g., U87-MG, LN18, or U251 cells) or patient-derived cells into immunocompromised mice, and these models retain human GBM features and can be used in therapy studies. Nevertheless, these models lack the proper immune environment; hence, tumor immunology research in such models cannot be performed. The syngeneic glioma transplantation model retains the authenticity of an immunocompetent host. Established mouse or rat C6, 9L and GL261 glioma cells that originate from tumors induced by methylnitrosourea or methylcholanthrene are injected into C57/B6 mice or rats, and the models are used to study the biology of glioma or new therapeutic agents. However, the established glioma cell line transplantation models do not reflect the heterogeneity of gliomas or the pathogenesis of the disease in human.

### Genetically engineered models

Molecular analyses of human gliomas have identified several mutations and alterations in the human glioma genome. The genetics and histology of xenograft models do not precisely reflect those of human glioma. Retroviral or transgenic glioma models involve manipulation of the mouse genome at the molecular level to create the molecular subtypes seen clinically. Approaches to generating mouse models of glioma include induction of loss-of-function (loss or inhibition of key tumor suppressors) or gain-of-function (expression of oncogenes) and somatic gene transfer by viral vectors. P53, PTEN, Nf1, and Rb are typically targeted tumor suppressors for the generation of mouse models of gliomas. V-Src, RAS and EGFRvIII are the most common oncogenes that have activating mutations and abnormal expression in gliomas.

In the Ctv-a mouse model, the transfer of PDGF-B to OPCs induces the formation of gliomas that resemble human WHO grade II oligodendroglioma 85. Mice transgenic for S100b-*verbB* and with p53 deletion can develop oligodendrogliomas with expression signatures similar to those of OPCs 86. The injection of VSV-G pseudotyped PDGF-IRES-Cre retrovirus into the rostral subcortical white matter of transgenic mice with floxed Pten and p53 induces proneural-type GBM 87. Mature neurons and astrocytes can also give rise to GBM tumors in mice. Injections of shNF1-shp53 virus into the cortex of synapsin I-Cre transgenic mice causes expression of Cre specifically in neurons, leading to the formation of gliomas 88. Similarly, CamK2a-Cre mice injected with the same virus, which targets mature neurons, develop tumors 88. In the hippocampus, subventricular zone, striatum or cortex, the majority of cells expressing GFAP are mostly differentiated astrocytes. When H-RasV12-shp53 virus is injected into these areas, all GFAP-Cre mice develop tumors 88

Many genetically engineered glioma models have been established based on gene knock-in or knock-out in embryonic stem cells. Experiments models with enhanced expression of potential oncogenes driven by a designated promoter can reveal their roles in tumor formation. GFAP promoter-driven overexpression of PDGFB in the brain lead to the development of glioblastoma in Trp53-null mice 89. S100β is glial-specific expressed and is expressed in both oligodendroglia and astrocytes early in brain development. All grades of oligodendrogliomas overexpress EGFR, and only high-grade tumors are found to have ink4a/arf deletion. Transgenic mice expressing *v-erbB*, a transforming allele of EGFR driven by the S100β promoter, develop low-grade oligodendroglioma, and mice with ink4a/arf or p53 heterozygous mutations develop high-grade tumors 90. However, the tumors of genetically engineered models based on embryonic stem cells greatly differ from human tumors, and the genetic manipulation can lead to significant developmental aberrations and can even be lethal to the animal before the tumor form.

Targeted expression of gene mutations is usually achieved through the Cre-lox system, which can be employed for knockout or forced expression of the target gene. Most commonly, transgenic Cre mice express the recombinase driven under the control of the GFAP promoter or Nestin (expressed in the neural progenitor cells) promoter. Transgenic mice harboring heterozygous loss-of-function mutations in Nf1 and p53 driven by GFAP-cre develop variable grade astrocytoma at 5-10 months. Mutation of PTEN causes accelerated tumor formation in this model and increases the grade of tumors. Between 15 to 40 weeks of age, 73% of the hGFAP-Cre; p53^lox/lox^; Pten^lox/+^ mice harbored grade III astrocytomas and GBMs 91. HGG exhibits strong RAS activation in humans. Activation of oncogenic K-ras in mouse glioneuronal precursor cells and adult subventricular zone cells with GFAP-Cre resulted in intermediate-grade, infiltrating glioma with 100% penetrance at 3 months. Cre recombinase-modified estrogen receptor ligand binding domain fusion protein (cre-ERT) translocates to the nucleus and initiates recombination after administration of tamoxifen. Inducible nestin-creERT mice to incorporate the tumor suppressor Nf1^flox/+^; p53^flox/flox^; Pten^flox/+^ or Nf1^flox/flox^; p53^flox/flox^ developed high grade astrocytomas after tamoxifen treatment 92.

How tumor cells co-evolve with the TME during tumorigenesis and tumor development (from the first gene mutation to tumorigenesis to tumor development) remains to be resolved, and one of the reasons is the lack of relevant models. Mosaic analysis with double markers (MADM) is a mouse genetic system that can be used to explore the dynamic interactions between tumor cells and other non-malignant cells in the TME. By design, MADM allows simultaneous gene knockout and fluorescent labeling of different genotype cells with different colors via Cre/loxP mediated mitotic recombination between two homologous chromosomes. MADM simultaneously and permanently generates sparse mutant cells with green fluorescent protein (GFP) and wild-type (WT) cells with red fluorescent protein (RFP) which are derived from the same precursor cell. The sibling red WT cells serves as an ideal internal control, facilitating detailed analyses of cellular aberrations of all lineages in the native environment 58. With these well-designed features, the MADM system has been applied to trace the cell origin of tumors at single-cell resolution. hGFAP-Cre/Nestin-Cre-induced and MADM-mediated sporadic concurrent inactivation of p53 and NF1 in neural stem cell transgenic mice lead to 100% high-grade astrocytoma/GBM at 5 months 58. The results indicated that OPCs are the cell of origin for glioma in this model. MADM-based genetic model in which sporadic Ptch1^+/-^, p53^+/-^ granule neuron precursors (GNPs) generated by Math1-Cre develop SHH-subtype medulloblastoma in two months 93. Math1-Cre faithfully labeled GNPs, enabling to study the establishment and evolution of the TME throughout tumorigenesis 93. Sparse tumor GNPs transdifferentiate into TuAstrocytes, which secrete IL-4 to polarize microglia and induce IGF1 secretion, which is critical for tumor progression 93.

## Single-cell RNA-seq in glioma

Single-cell RNA sequencing (scRNA-seq) enables the examination of the heterogeneity of tumor cells and the TME, shedding light on the interaction of the TME with tumor cells. The scRNA-seq of gliomas measures gene expression at the single-cell level, thereby uncovering the heterogeneity and evolutionary trajectory of the TME and tumor cells and providing molecular insight into precision therapy. We next briefly discuss what scRNA-seq reveals about the heterogeneity of tumor cells, TME cells and their phenotypic alterations in the TME.

### Single-cell RNA-seq of tumor cells

In recent years, scRNA-seq has been used in studies such as The Cancer Genome Atlas (TCGA) to map the genetic landscape and expression signature of tumors and TME cells, thereby identifying driver mutations and tumor subtypes on specific transcriptional profiles. A large cohort study with single cell analysis provides a general framework for understanding the genotype, phenotype and TME composition of coupled tumor cells.

The scRNA-seq profiles were extracted from IDH-mutant astrocytoma (IDH-A) and oligodendroglioma (IDH-O) to decipher the differences in tumor cell genotypes and phenotypes between the two tumor subtypes. The IDH-A and IDH-O share a similar developmental hierarchy and spectrum. Tumor cell morphological and histopathologic differences between the two subtypes can be explained by distinct genetic events and TME composition 94. Tumor pathological grade is associated with increased proliferation of glioma cells, suppressed differentiation of tumor cells, and alignment with highly polarized tumor associated macrophages/microglia 94.

GBM can be classified into proneural, neural, classical, and mesenchymal subtypes based on bulk gene expression data 95. By leveraging the cellular resolution of the technique, scRNA-seq highlights intratumoral heterogeneity in primary glioblastoma. Analysis shows that four subtypes could be observed in individual patient tumors 96. Another scRNA-seq analysis also demonstrated that malignant cells in each glioblastoma sample exist in four main cellular states including (1) neural-progenitor-like (NPC-like), (2) oligodendrocyte-progenitor like (OPC-like), (3) astrocyte-like (AC-like), and (4) mesenchymal-like (MES-like) states, and exhibit distinct patterns of distribution in the TME 97. The relative proportion of each cellular type is attributed to genetic alterations in CDK4, NF1, EGFR, and PDGFRA 97. ScRNA-seq of GBM revealed extensive genetic heterogeneity at the transcriptional level. Expression and/or mutational heterogeneity of RTKs exists among individual glioblastoma cells and may affect treatments targeting receptor immunogenicity or RTK signaling 96. In addition, intratumoral heterogeneity is associated with prognosis. In the proneural subtype, increased intratumoral heterogeneity is relevant to decreased survival 96. By comparing the differences before and after treatment, scRNA-seq could also help us understand previous therapeutic failures and explore potential therapeutic targets.

### Single-cell RNA-seq of the TME

Molecular and genetic variation in cancer cells may translate to phenotypic and functional variation in the TME. Emerging evidence suggests the close association between the molecular profile of glioma tumor cells and their surrounding microenvironment, and thus this heterogeneity in molecular state may also be imprinted into microenvironmental heterogeneity within tumors.

Combined flow cytometry sorting and scRNA-seq analysis were used to investigate the immune and non-immune components of GBM with single-cell resolution, and populations were analyzed longitudinally during initiation and progression. As GBM progresses, the immune cell composition changes. The immune microenvironment during the early stages of GBM tumorigenesis involves the transformation of "M1"-like pro-inflammatory microglia into centrally infiltrating "M2"-like tumor-promoting macrophages during the later stages 98. These transitions coincide with the collapse of the BBB and the explosive growth of EGFR+ cancer cells 98. ScRNA-seq was used to distinguish blood-derived macrophages and microglia under malignant and non-malignant conditions. Müller et al. identified phenotypic differences among TAMs of different lineages by scRNA-seq analysis of IDH-wt GBM 99. TAMs derived from blood preferentially express immunosuppressive cytokines compared with microglia. A significant amount of blood-derived TAMs infiltrated pretreatment gliomas, which varied with tumor subtype and compartment 99. Since blood-derived TAMs are more closely associated with poor prognosis, specifically targeting TAMs from immunosuppressive blood sources appears to be a better therapeutic strategy.

The scRNA-seq of endothelial cells (ECs) from human GBM tumor core and adjacent tissues provides a molecular perspective on the heterogeneity of the human BBB and its metabolic alterations in glioblastoma. The ECs in tumors are associated with altered metabolic transcriptome profiles characterized by increased expression of genes involved in glycolysis, citric acid cycle and oxidative phosphorylation 100. Understanding the heterogeneity of the BBB phenotype is also crucial to develop rational treatment regimens and optimize drug delivery.

Multifocal samples are considered excellent models for studying the natural evolution of GBM, as they may represent different stages of tumor development. Wang et al. conducted a comprehensive analysis of eight multifocal IDH wild-type primary GBMs by sequencing 61,062 single cells 101. By analyzing 4 pairs of GBM lesions in different regions, two lesions were found to be of the same origin but with different copy number changes. There is an underlying evolutionary sequence order between the two lesions. Further single-cell sequencing analysis revealed that the natural evolution of GBM may be accompanied by widespread gene activation and the acquisition of a module consisting of 12 genes, and the genes in the module were considered a natural evolutionary signature (NES) 101. NES is significantly associated with tumor development, clinical outcome, and tumor cell status. Integrating scRNA-seq of multifocal GBMs identified FOSL2 as one of the hallmarks of higher expression in older lesions that may be involved in tumor evolution 101. Furthermore, the hypoxic response was significantly associated with NES, possibly involved in NES transition through activation of the HIF1A-FOSL2 axis 101. NES-high tumor cells recruit and polarize BMDMs by activating the FOSL2-ANXA1 axis to suppress T cell activity 101. Polarized TAMs can also upregulate CCL2 to induce tumor cell migration 101. This study further highlights the significance of single cell sequencing in investigating the evolution of glioma.

Using single-cell sequencing data, we are able to identify different cell subtypes and further analyze the evolutionary relationships between each subtype of tumor cells as well as TME cells. This is crucial for the successful development of precise therapy for glioma.

## Future clinical application

Although there has been great progress in multimodal comprehensive treatment for gliomas, including surgery, radiotherapy, and systemic therapy (chemotherapy, targeted therapy), the outcomes are still not satisfactory. The immune cells of malignant glioma can “harmonize” with tumor cells and other components of the TME, thus forming a microenvironment conducive to the proliferation of glioma cells. Targeting tumor cells and their “partners” has become an attractive strategy in precision medicine (Table 1).

### TAM therapy

Targeting TAMs is a promising efficacious therapeutic strategy. Currently, approaches include inhibition of TAM recruitment, TAM depletion and TAM re-education.

The first approach is to inhibit the accumulation of macrophages or microglia in tumors by targeting chemoattractants and/or their receptors. Preclinical data show that a CCL2-neutralizing antibody prolongs the survival in mouse and human glioma xenograft models by inhibiting TAM infiltration 102. In addition, blockade of CCL2/CCR2 signaling enhances the efficacy of temozolomide 102 or immune checkpoint inhibitors 103 in GBM mouse models.

Alternative strategies include reducing the population of immunosuppressive TAMs or converting TAMs from a tumor-promoting phenotype to a tumor-inhibiting phenotype. CSF-1/CSF-1R is the essential signaling pathway for macrophage survival and proliferation, but the CSF-1R inhibitor BLZ945 suppresses glioma growth and progression by altering macrophage polarization, but not by depleting macrophages 104, and improves the survival rate of glioma mice 105. However, resistance to long-term BLZ945 treatment ultimately develops in a manner driven by the reprogramming of TAMs in the TME through the IGF-1/PI3K pathway 105. PLX3397 (another CSF-1R inhibitor) markedly suppresses glioma cell proliferation and invasion and reduces the grade of malignancy in a PDGF-B-driven proneural glioma mouse model 106. In addition, PLX3397 enhances the sensitivity of glioma cells to tyrosine kinase inhibitors by re-educating TAMs in preclinical combination trials 106. Surprisingly, PLX3397 shows no survival benefit in clinical trials of GBM patient, despite showing good tolerability and adequate BBB penetration 107. The SIRPα-CD47 signaling axis is important for macrophage phagocytosis. Recently, research demonstrated that anti-CD47 strategies show promising antitumor effects in various gliomas, including GBM 108 and pediatric glioma 109, by increasing TAM-mediated phagocytosis of macrophages.

### Immune checkpoint blockade therapy

Glioma cells can evade immune surveillance through activation of immune checkpoint ligands such as PD-1, CTLA-4, and IDO. Numerous studies over the past decade have reported that preclinical trials have succeeded in the treatment of gliomas. CTLA-4 blockade prolongs survival in 80% of treated animals and enhances antitumor immunity by increasing the proliferation of CD4+ T cells without affecting Treg suppressive function 110. The combination of PD-1 blockade and localized radiation therapy increases tumor infiltration by cytotoxic T cells and decreases Tregs, which results in long-term survival in mice with orthotopic glioma 111. In a glioma mouse model, 100% of mice survive long-term following IDO, CTLA-4, and PD-L1 blockade therapy, and tumor-infiltrating of Tregs is decreased 112. However, in CheckMate 143 (NCT02017717), the first clinical trial of PD pathway inhibition in GBM, nivolumab failed to prolong the OS of recurrent GBM patients compared with the anti-VEGF antibody bevacizumab 113. Surprisingly, a recent clinical trial showed that anti-PD-1 immunotherapy induced local and systemic immunomodulatory treatment effects in recurrent glioblastoma 114. In addition, a phase II study (NCT02337491) including 80 patients with recurrent glioblastoma reported that pembrolizumab (another PD-1 monoclonal antibody) alone or with bevacizumab therapy does not significantly improve OS 115. Intracerebral administration of nivolumab and ipilimumab (CTLA-4 monoclonal antibody) was well-tolerated and associated with encouraging OS in recurrent glioblastoma patients with maximal safe resection 116.

### Dendritic cell vaccines

Dendritic cell vaccines (DCVs) are also gaining substantial clinical attention because they have considerable promise in the treatment of glioma, and several preclinical and clinical trials are underway. The first two studies used DCVs to treat glioma patients, and this treatment safely induced immune responses 117 and enhanced the median survival of patients by improving tumor-specific T-cell activation, infiltration, and/or function 118. In addition, 31 other studies have been published from 2001 to 2018 detailing the treatment of GBM with DCVs. Currently, there are three phase III trials using DCV treatment for GBM registered at ClinicalTrials.gov (#NCT00045968, #NCT01759810, # NCT02546102). Recent studies have also sought to identify factors that enhance the efficacy of DCVs. One study reported that combining a DCV with temozolomide could enhance antitumor immunity by increasing tumor-specific immune responses in glioma 119. Gene therapy targeted to Flt3L and HSV1-TK in combination with a DCV can improve antitumor immunity, therapeutic efficacy and long-term survival by modifying the brain TME in a syngeneic glioma model 120. Another study identified that the AKT inhibitor MK2206, the DNA-PK inhibitor NU7441, and the MEK inhibitor trametinib increase the ability of a DCV to activate and expand tumor-reactive T cells and augment antitumor activity in glioblastoma mice 121. Pre-conditioning the vaccine site with a potent recall antigen (e.g., tetanus/diphtheria toxoid) significantly enhances DC migration bilaterally and improves the survival of glioblastoma patients, and this phenomenon is dependent on the chemokine CCL3 in murine models 122. The combination of DC immunotherapy with hypericin-based photodynamic therapy to induce immunogenic cell death (ICD) enhances anti-tumor responses and improves OS in preclinical glioma models 123.

### Targeting the vasculature

Angiogenesis is a hallmark of glioblastoma and remains an important therapeutic target in its treatment. Targeting blood vessel-related factors, particularly VEGF and VEGFR, has been an attractive strategy in gliomas. Bevacizumab, a humanized monoclonal antibody against VEGF, is currently approved for recurrent glioblastoma. In one study, after treatment with bevacizumab combined with other chemotherapy agents, edema was decreased and progression-free survival (PFS) and OS were increased in patients with recurrent glioma 124. However, the use of bevacizumab does not improve OS and PFS in newly diagnosed glioblastoma 125. This class of therapy causes adverse events, including hypertension, proteinuria, poor wound healing, thrombosis, convulsion, neutropenia, and reduced cognitive function, which impair the patient's quality of life over time.

An abnormal vasculature creates an abnormal microenvironment and blocks the effective delivery of therapeutic agents to tumors. Vascular normalization therapy supports physiological angiogenesis to improve the TME (reduce hypoxia, improve perfusion and drug delivery) and thus may be an alternative strategy for tumor therapy. AZD2171 (cediranib), a pan-VEGF receptor tyrosine kinase inhibitor, normalizes the structure and function of the tumor vasculature, alleviating edema and increasing tumor perfusion, which is associated with prolonged PFS and OS in recurrent glioblastoma patients 126. These data identify potential new targets for recurrent glioblastoma treatment.

## Conclusion

Gliomas are among the leading causes of cancer-related deaths and have poor prognosis and a high rate of recurrence. In recent years, tumors have been recognized as complex tissues that recruit TME cells that also play key roles in every aspect of tumor development. Increasing evidence indicates that tumor-TME cell interactions lead to the co-evolution of tumor cells and the surrounding environment, which is critical in glioma initiation and development. The development of appropriate mouse models could improve the understanding of the mechanisms underlying these interactions and the identification of new therapeutic targets for treatment. Considering the high abundance and genetic stability of cells in the TME, TME-targeting therapy has emerged as a promising strategy. TAM-directed therapies combined with immune checkpoint blockade therapy and/or DCVs could enhance the antitumor effect of tumor-infiltrating lymphocytes and then phenotypically shift the TME from a “cold” to a “hot” environment. Recently, the results of an encouraging phase 3 prospective externally controlled cohort trial (NCT00045968) indicated that an autologous tumor lysate-loaded DCV combined with temozolomide chemotherapy extended OS in both newly diagnosed and recurrent GBM 127. In the future, clinical translation of treatment strategies to re-educate or manipulate factors in the abnormal TME in gliomas should be achieved.

## Figures and Tables

**Table 1:** Clinical trial of microenvironment-targeting therapies in brain tumors

Drug	Target	Phase	Tumor types	Study Identifier
**BLZ945**	CSF-1R inhibitor	I/II	GBM/rGBM	NCT02829723
**PLX3397**	CSF-1R inhibitor	II	rGBM	NCT01349036
**Nivolumab**	Anti-PD-1	II/III	rGBM	NCT02017717
**Pembrolizumab**	Anti-PD-1	II	rGBM	NCT02337491
**Ipilimumab**	anti-CTLA-4	I	rGBM	NCT03233152
**DCVax-L**	dendritic cell vaccine	III	GBM/rGBM	NCT00045968
**Bevacizumab**	anti-VEGF-A	approved	Recurrent glioma	
**Bevacizumab**	anti-VEGF-A	III	Newly diagnosed GBM	NCT00884741
**Cediranib**	inhibits VEGFR	II/III	rGBM	NCT00777153

## Abbreviations

ADAM10: a disintegrin and metalloproteinase domain-containing protein 10
AMPAR: α-amino-3-hydroxy-5-methyl-4-isoxazole propionic acid receptors
BBB: blood-brain barrier
BBTB: blood-brain tumor barrier
BMDMs: bone marrow-derived macrophages
BMX: bone marrow and X-linked
CAMK2A: calcium/calmodulin dependent protein kinase II alpha
CLNs: cervical lymph nodes
CNS: central nervous system
CSF-1: colony stimulating factor-1
CTLA-4: cytotoxic T lymphocyte 4
DCs: dendritic cells
DCVs: dendritic cell vaccines
ECM: extracellular matrix
EGF: epidermal growth factor
FGF: fibroblast growth factor
FGL2: fibrinogen-like protein 2
GBM: glioblastoma multiforme
GDF-15: growth differentiation factor 15
GFAP: glial fibrillary acidic protein
GFP: green fluorescent protein
GNPs: granule neuron precursors
GSCs: glioma stem cells
HGF: hepatocyte growth factor
HGG: high-grade gliomas
HMGB1: high mobility group box 1
IDO: Indoleamine 2,3-dioxygenase
IFN-γ: Interferon gamma
IGF-1: insulin-like growth factor 1
IRES: internal-ribosomal entry sites
LYVE-1: lymphatic endothelial hyaluronic acid receptor 1
MADM: Mosaic Analysis with Double Markers
MAPK: mitogen-activated protein kinases
MHC: major histocompatibility complex
MICA: class I-related chains A
MLV: Meningeal lymphatic vessels
MMP-2: matrix metalloproteinase-2
NETs: neutrophil extracellular traps
NF1: Neurofibromatosis type 1
NF-κB: nuclear factor kappa-B
NK: natural killer
NKG2D: NK group 2 member D
NLGN3: neuroligin-3
OPCs: oligodendrocyte precursor cells
OS: overall survival
PD-1: programmed cell death protein 1
PDGF-B: platelet-derived growth factor B
PD-L1: programmed cell death ligand 1
PFS: progression-free survival
PI3K: phosphatidylinositol-3-kinase
Prox1: prospero-related homeobox 1
PTEN: phosphatase and tensin homolog
RAGE: the receptor for advanced glycation end-products
RANKL: NF-κB ligand
RFP: red fluorescent protein
S1P1: Sphingosine-1-phosphate receptor 1
scRNA-seq: single-cell RNA sequencing
SDF-1: stromal-cell derived factor-1
SHH: Sonic hedgehog
SIRPα: Signal regulatory protein α
STAT-3: signal transducer and activator of transcription 3
STI1: stress-inducible protein 1
TAAs: tumor-associated astrocytes
TAMs: tumor associated myeloid cells
TGF-β: transforming growth factor-β
Tim-3: mucin-domain-containing molecule 3
TINs: tumor-infiltrating neutrophils
TLR2: Toll-like receptor 2
TME: tumor microenvironment
TNF-α: tumor necrosis factor
Tregs: regulatory T cells
TRM: tissue-resident microglia
ULPB-2: UL16 binding protein
VEGF: vascular endothelial growth factor
VSVG: virus G glycoprotein
WHO: World Health Organization
WT: wild-type
